# Flexural Response of Functionally Graded Rubberized Concrete Beams

**DOI:** 10.3390/ma17081931

**Published:** 2024-04-22

**Authors:** Abdulrahman S. Albidah, Abdulaziz S. Alsaif

**Affiliations:** Civil Engineering Department, College of Engineering, King Saud University, P.O. Box 800, Riyadh 11421, Saudi Arabia

**Keywords:** flexure, beams, waste rubber, steel fiber, functionally graded

## Abstract

Recycling rubber and/or steel fiber components of waste tires in construction applications is a venue for maximizing the recycling rate of these items. Additionally, it supports the move towards producing sustainable construction materials and conserving natural resources. Previous research explored the viability of employing recycled waste rubber particles as an alternative for natural aggregate. Despite the adverse effect of rubber on the mechanical properties of concrete (e.g., lower compressive strength), it produces several advantages, including excellent dynamic and ductility properties, which can be utilized in structural members critical to dynamic loads, e.g., blasts, earthquakes, and impacts. In an effort to expand the adoption of waste rubber in concrete beams and to eliminate key concerns associated with the degradation of their flexural behavior, the functionally graded (FG) beams concept was utilized. The present investigation comprised the testing of five beams using a four-point bending configuration. Plain concrete, rubberized concrete (RuC), and steel-fiber reinforced rubberized concrete (SFRRuC) beams were cast along with FG beams arranged in two layers. The top layer of the FG beams comprised plain concrete, while the bottom layer consisted of RuC or SFRRuC. Experimental findings indicated that the flexural behavior of the FG beam with layers of SFRRuC and plain concrete exceeded the flexural strength, displacement ductility ratio, and toughness performances of the plain concrete beam by 9.9%, 12.9%, and 24.4%, respectively. The moment–curvature relationship was also predicted for the tested beam and showed an excellent match with the experimentally measured relationship.

## 1. Introduction

Urbanization and the increased demand for the various means of transportation have significantly increased the accumulation of post-consumer tires. The amount of discarded tires produced in China was approximately 14.5 Mton in 2019 [1]. However, poor waste management of post-consumer tires is a serious issue, with the potential for the occurrence of accidental fires and associated emissions aggravated by the tires’ high flammability [2], the occupation of excessive landfill space, and the facilitation of the breeding of mosquitoes with the consequent spread of infectious diseases [3]. Innovative and sustainable solutions for effectively managing the disposal of waste tires are therefore needed.

Recycling the rubber and/or steel fiber components of waste tires in construction applications presents an opportunity for maximizing the recycling rate of rubber tire waste. Moreover, it supports the move towards sustainable production of construction materials and conserving natural resources. Significant research has been undertaken for the viability assessment of reclaimed rubber particles to substitute partially coarse aggregate, fine aggregate, or a blend of both. The addition of recycled rubber into concrete mixtures has been associated with reductions in the compressive strength [4,5,6], elastic modulus [7,8], and mix workability [4,7]. The mechanical properties’ degradation has primarily two main causes: (i) the deficiency in bond between rubber and the cementitious matrix, and (ii) the large disparity in stiffness between the rubber and other constituents in the concrete mix [3]. However, rubberized concrete (RuC) also offers several advantages: reduced unit weight [9,10], better noise and heat insulation [11,12], and improved cracking resistance [13,14]. Additionally, the incorporation of rubber in the concrete mix produces excellent dynamic and deformability properties which can be exploited in structural members subject to dynamic loads, e.g., blasts, earthquakes, and impacts. Research has shown that RuC has an improved toughness [15,16], energy dissipation capacity [17], ductility [14,18], damping [19,20], fatigue resistance [21], and impact resistance [5,22].

Despite the abundant research carried out investigating the mechanical characteristics of RuC, studies of the behavior of structural concrete members cast with RuC are generally limited [3]. Research has been carried out to assess the flexural performance of RuC beams [2,23,24,25,26,27]. Mendis et al. [23] explored the flexural behavior of twelve rubberized concrete beams cast in two groups. The first group targeted compressive strengths between 30 and 35 MPa, while the other group targeted compressive strengths between 40 and 45 MPa. The substitution percentages of fine aggregates with crumb rubber ranged from 5.3% to 21.1% in the concrete mixes. It was observed that reinforced concrete beams cast with RuC with similar compressive strengths also possessed similar ultimate flexural strengths, cracking moment, and load-deflection behavior, irrespective of the mix proportions and rubber content. The design guidelines in three design codes for conventional concrete structures were also found to be appropriate for predicting the cracking moment and ultimate flexural strength for crumb rubber concrete beams, to an equivalent degree of accuracy.

Ismail and Hassan [24] investigated the flexural response for twelve beams prepared with natural aggregate replaced by 0–40% vol/vol crumb rubber for self-consolidated concrete and 40–50% vol/vol crumb rubber for vibrated concrete. They observed that increasing the crumb rubber content resulted in an increased beam curvature at service load (i.e., improved deformation capacity). The substitution of virgin aggregates by up to 20% of crumb rubber resulted in an improved curvature ductility. Additionally, it was generally noticed that increasing the crumb rubber quantity limited crack widths and led to a higher number of cracks. Hassanli et al. [25] assessed the flexural response of four beams subjected to half-cyclic loading until failure. The beams were cast with NaOH surface-treated crumb rubber particles replacing natural aggregate in proportions of 0, 6%, 12%, and 18%. They noticed that with an increased rubber content the failure mode was dominated by flexure, with a growth in the number of cracks and a reduction in the crack width. The flexural capacity declined by 2.3%, 1.7%, and 6% for beams incorporating crumb rubber replacing natural aggregates by 6%, 12%, and 18%, respectively. An experimental program by Hall and Najim [26] comprised testing plain and self-compacting RuC beams. Crumb rubber particles pre-coated with mortar were utilized to replace 9% of coarse and fine aggregates in a plain mix and 7% in a self-consolidating mix. They reported that the initial crack deflection was generally higher for beams with rubber content, suggesting greater absorption of kinetic energy before the elastic limit. Ismail et al. [27] tested four beams in flexure, with crumb rubber replacing natural fine aggregates. All four beams were cast using self-consolidating concrete, with rubber substitution percentages of 0, 5%, 10%, and 15%, respectively. The addition of rubber caused a slight decrease in flexural strength, an improvement in ductility, and reduced crack widths.

Research was also conducted on steel fiber reinforced rubberized concrete (SFRRuC) beams [2,28]. Eisa et al.’s [2] experimental program included tests on beams containing crumb rubber particles substituting fine aggregates by 5%, 10%, 15%, and 20% with and without steel fibers. The beams were intended to be dominated by flexure by providing sufficient shear reinforcement. It was concluded that beams with 20% crumb rubber reduced their flexural capacity by up to 20% in comparison to beams having no rubber. However, adding steel fibers to the RuC mix with 20% rubber improved its behavior and reduced its flexural strength by only 9%. Karthikeyan et al. [28] studied the flexural performance of seven SFRRuC beams fabricated with sand-coated coarse rubber replacing 2.5%, 5%, and 7.5% of natural coarse aggregates. For each replacement percentage, two beams were cast with 0.5% and 1% of steel fibers. Beams containing 1% steel fibers and 7.5% rubber were shown to have enhanced flexural strength, deformation capacity, ductility, and energy capacity at rates of 20.8%, 107.5%, 40%, and 83.1%, respectively, compared to a control beam without rubber and steel fibers.

Functionally graded materials (FGMs) comprise two or more materials that have different properties [29,30]. The purpose of FGMs is to optimize the use of materials based on their properties; for example, high-strength concrete is placed in the extremely stressed compression zone, while lower-strength concrete is positioned in the extreme tension fiber zone [31]. Torelli et al. [32] pointed out that, in the literature, the key drivers for utilizing FGMs are reported to be cement reduction, improved post-fracture behavior, weight minimization, and improved durability of the concrete elements.

Studies have explored the effectiveness of FGMs on compressive and bending strengths [33,34,35]. Bajaj et al. [33] investigated the behavior of FG compressive cubes and flexural prisms cast in two layers, one made of traditional concrete, and the other of high-volume fly ash concrete with the fly ash replacing 20%, 35%, and 55% of the cement content. The depth of the interface between the two materials was a primary variable. The improvement in the FG compressive and flexural strengths was approximately 12.9% and 3.6%, respectively. Liu et al. [34] investigated FGMs comprising conventional concrete and concrete containing recycled aggregate with and without steel fibers. Compressive and bending tests concluded that FG concrete has a strong potential in terms of its structural design. Choudhary et al. [35] explored the influence of rubber fibers from waste tires on FG concrete. The fibers replaced natural fine aggregates at rates from 5% to 30% in an incremental step of 5%; the test parameters were flexural strength, compressive strength, water absorption, and water permeability. The flexure and compressive strength samples were cast in two layers of equal depth, in which the base layer consisted of a rubber fiber mix and the upper layer was a plain concrete mix. It was generally observed that FG RuC samples had better mechanical and durability characteristics than samples cast with only rubberized fiber concrete.

Studies have also been performed to evaluate the impact of FGMs on beam behavior [29,31,36,37,38]. Palaniappan et al. [29] investigated FG concrete beams in which conventional concrete was cast in the compression region while the concrete in the tension region incorporated various percentages of fly ash or red mud as a cement replacement. It was noticed that the FG beam exhibited better strength and durability than the conventional concrete beam. Maalej et al. [36] tested reinforced FG concrete beams to evaluate their resistance to corrosion and their overall structural response when exposed to accelerated corrosion. A layer of ductile fiber-reinforced cementitious composite material was cast around the main flexural reinforcement for corrosion protection. Their resistance to steel corrosion was found to be notably higher than for conventional concrete beams. A study by Pratama et al. [31] involving tests of flexural beams having two/three layers of concrete with compressive strengths of 25–30 MPa showed insignificant variation in the flexural capacity for the control and FG beams. Naghibdehi et al. [37] explored the flexural performance of FG beams in which steel and polypropylene fibers were utilized in separate layers of unreinforced concrete. The fiber content varied from 0% to 2%. It was noticed that the flexural strength was higher when steel fibers were distributed over the whole cross-section than when two types of fibers were used in individual layers. Sharaky et al. [38] investigated the flexural behavior of eight FG rubberized concrete beams and one control beam with plain concrete across the entire cross-section. For the eight FG beams, the cross-section was divided into three equal layers. The first group of beams consisted of plain concrete in the top and bottom layers, whereas the middle layer contained rubberized concrete with a rubber content of 30, 50, or 80%. The second group had beams with plain concrete in the top layer, rubberized concrete (30% crumb rubber) in the middle layer, and rubberized concrete (10, 20 or 30%) in the bottom layer. The third group had beams with plain concrete in the top layer, rubberized concrete (30% crumb rubber) in the middle layer, and steel fiber reinforced concrete in the bottom layer. 

Based on the reviewed literature, it is clear that, to date, the investigation of the utilization of rubber in structural members has generally been limited to fine rubber particles substituting fine aggregate at rates of mostly up to 20%. The avoidance of higher replacement levels has evidently been to minimize the strength reduction. Furthermore, with the exception of research by Sharaky et al. [38], FGMs have never been used in combination with flexural beams containing rubber tire particles. However, the authors hypothesized that adopting the concept of FGMs in casting beams would allow the employment of a large content of fine and coarse rubber particles without extra cost and without reducing the beams’ capacity, while achieving all the merits of RuC.

This research experimentally studied the flexural response of five concrete beams. Control and FG beams were cast with plain concrete, RuC, and SFRRuC. Coarse and fine waste tire rubber were employed to substitute 20% of both the coarse and fine natural aggregates by volume. The steel fibers were a mixture of manufactured and recycled tire steel fibers. A moment–curvature analysis was also predicted to validate the experimental results.

## 2. Experimental Investigation

### 2.1. Mix Preparation and Material Characteristics

**Figure 1:**
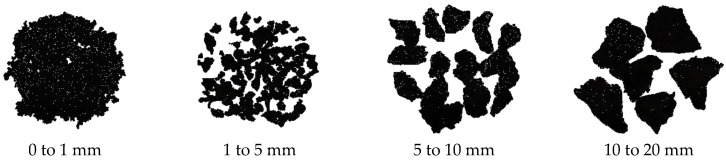
Photographs of the waste tire rubber.

**Figure 2:**
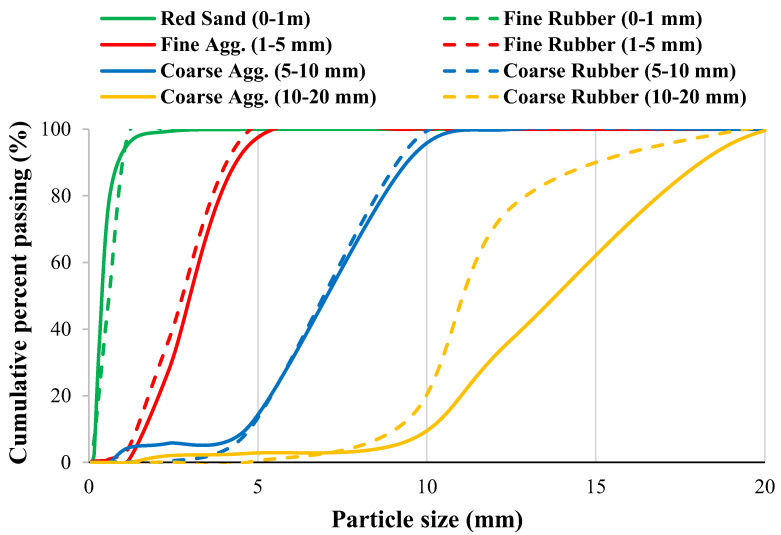
Natural and recycled rubber aggregates size distributions.

Three concrete mixes were prepared for the present investigation: normal concrete, RuC, and SFRRuC. The normal concrete mix (mix P) was prepared using Portland cement Type-1, natural coarse and fine aggregates, superplasticizer, and water. The natural coarse aggregate was a blend of 5 to 10 mm and 10 to 20 mm limestone and the fine aggregate was 1 to 5 mm crushed limestone and red sand. Rubberized concrete (mix Ru) resembled the normal concrete mix, albeit with coarse and fine natural aggregates that were partially substituted by 20% (by volume) of the recycled rubber tire of corresponding particle size (photographs in Figure 1). The distribution of various aggregate sizes and types adopted are presented in Figure 2. The third mix (RuSF) was identical to mix Ru in all aspects except that it included a mixture of 20 kg/m^3^ manufactured steel fibers and 20 kg/m^3^ steel fibers from recycled tires. The manufactured steel fibers were the hooked-end type, measuring 60 mm in length and 0.75 mm in diameter. The recycled steel fibers had lengths ranging from 10 to 60 mm and diameters < 0.3 mm. It is worth mentioning that both recycled rubber and recycled steel fibers were obtained from mechanical shredding of waste tires by local recycling company. Additionally, both materials were used without any cleaning process or pre-treatment to enhance their cost effectiveness when incorporated in the concrete mix. 

Various mix proportions are listed in Table 1. The superplasticizer dosage was increased for the mixes Ru and RuSF (see Table 1) to compensate for the workability loss owing to the inclusion of rubber and/or steel fibers.

Deformed steel bars with 8 and 12 mm diameters were employed for the flexural and transverse steel of the beams. Table 2 lists the mechanical characteristics of the steel rebars.

### 2.2. Description of Test Specimens

Five reinforced concrete slender beams of similar geometry and steel reinforcement (longitudinal and transverse) were cast and tested until failure. All specimens were manufactured with a typical span length of 1500 mm, width of 120 mm, and depth of 180 mm. The bottom and top longitudinal steel comprised two 12 mm and two 8 mm diameter bars, respectively. The shear stirrups were of 8 mm diameter steel spaced at 100 mm centers, designed such that the total shear resistance of the beam exceeded the flexural capacity to guarantee purely flexural beam response. The concrete cover had a thickness of 20 mm.

**Figure 3:**
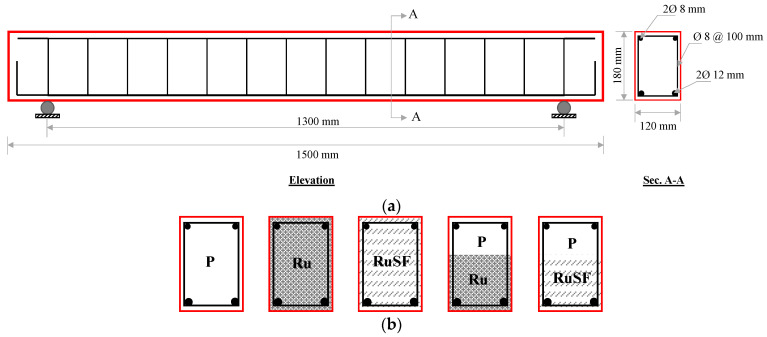
Beam geometry and steel details: (**a**) elevation and typical cross-section; (**b**) cross-sections showing the concrete type adopted for the various beams.

The only variable in this test program was the concrete type adopted to fill the beam cross-section. The first three beams designated as P, Ru, and RuSF were cast using plain concrete, RuC, and SFRRuC, respectively. The other two beams were cast adopting the functionally graded material concept. The fourth beam Ru + P was cast in two layers: the lower layer (representing 60% of the beam cross-section) consisted of RuC; the upper layer was cast with plain concrete. The fifth beam RuSF + P was similar to the fourth beam in all respects, except that the lower layer was cast with SFRRuC. Details of the beam’s reinforcement, geometry, and materials are summarized in Table 3 and Figure 3.

The functionally graded (FG) beams were cast to optimize the cross-sectional performance while utilizing RuC or SFRRuC. Due to the reduced mechanical properties of RuC exhibited by rubber addition, the RuC/SFRRuC was concentrated around the beam areas subjected to tensile stresses, as the tensile strength contribution of concrete to flexural behavior is generally negligible. Considering the critical role of the concrete compressive strength in determining the beam flexural capacity, relatively high compressive strength plain concrete was cast around the compressive stress zone. The 60/40 proportioning of the beam cross-section was made in light of preliminary sectional analysis to guarantee that plain concrete was employed in the entire compressive stress zone.

### 2.3. Casting and Curing Method

For FG beams, the casting was first made for the bottom layer filling 60% of the beam cross-section. Proper compaction and vibration were then done prior to casting the upper layer, which was carried out while the lower layer was in the fresh state. The duration between the completion of casting for the lower layer and the start of casting for the upper layer was within 30–40 min. The top layer was also vibrated and its surface was finished. 

Concrete cylinders measuring 100 mm diameter and 200 mm height were cast for each type of concrete mix and used to assess the compressive stress–strain behavior and the splitting tensile strength. After completion of pouring the beams and cylinders, they were wrapped in damp burlap in the ambient laboratory conditions (24 ± 2 °C) for three days. They were then demolded and remained wrapped in damp burlap in ambient laboratory conditions until the testing day.

### 2.4. Experimental Setup and Instrumentation

**Figure 4:**
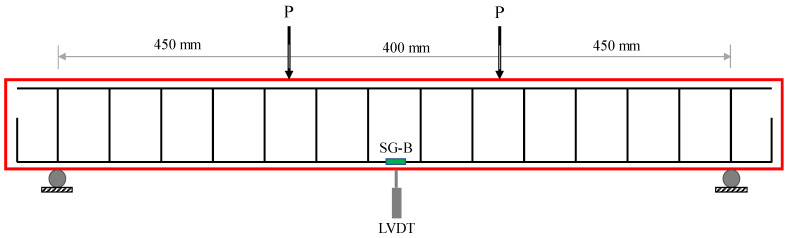
Typical setup for the investigated beams.

Four-point bending configuration was utilized to test the flexural behavior for all beams. The supports and loading points of the beam were rigid steel cylinders. The supports were placed such that the over hanged length was 100 mm from each ends, leading to an effective span length of 1300 mm. The loading points were 400 mm apart. The load was increased at a rate of 0.5 mm/min in a displacement-controlled mode until reaching the maximum load of the beam. After reaching the maximum load, the loading rate was doubled. The lower loading rate in the early loading stage was to allow more time for identifying and marking cracks and making observations. Linear variable differential transducers (LVDTs) were instrumented at the mid-span to measure the vertical displacement. Strains at the mid-length of the tensile steel were measured using steel strain gauges. Beam curvature was evaluated during the test using two lateral LVDTs positioned near the bottom and top of the beam within the pure bending zone. Instrumentation for the flexural test is demonstrated in Figure 4. The stress–strain behavior and splitting tensile strength were obtained following the ASTM C39/C39M [39] and ASTM C496/C496M [40], respectively.

## 3. Results and Discussion

### 3.1. Mechanical Properties of Concrete

#### 3.1.1. Stress–Strain Relationship

**Figure 5:**
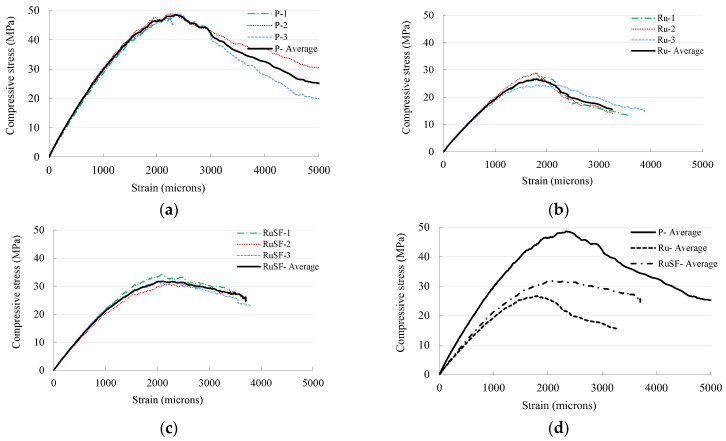
Stress–strain curves for mixes: (**a**) P; (**b**) Ru; (**c**) RuSF; and (**d**) all mixes.

Figure 5 shows the stress–strain relationship of the three mixes (P, Ru, and RuSF). Triplicate samples were evaluated and plotted for each concrete mix along with the average curve. Figure 5a–c show that the stress–strain curves were generally consistent for all specimens of each mix. The average compressive strength of the plain concrete at 28 days was 48.4 MPa, which decreased by 44.2% when rubber was added. The inclusion of steel fibers to the RuC reduced the decrease to 33.5%. Similarly, the inclusion of rubber, either with or without steel fibers, caused a decrease in the elastic modulus and strain at peak strength, which matched the trend observed for the compressive strength as provided in Table 4. The reduction in the compressive strength due to the addition of rubber was expected in light of previous research. This, in fact, motivated the authors to avoid substituting natural aggregates (both fine and coarse) by more than 20%, as an additional increase in rubber content can lead to a huge drop in the compressive strength, reaching more than 80%, as observed in tests by Alsaif et al. [8]. Although the presence of steel fibers partially compensated the drop in the compressive strength, its contribution to the compressive strength is still limited.

#### 3.1.2. Splitting Tensile Strength

**Figure 6:**
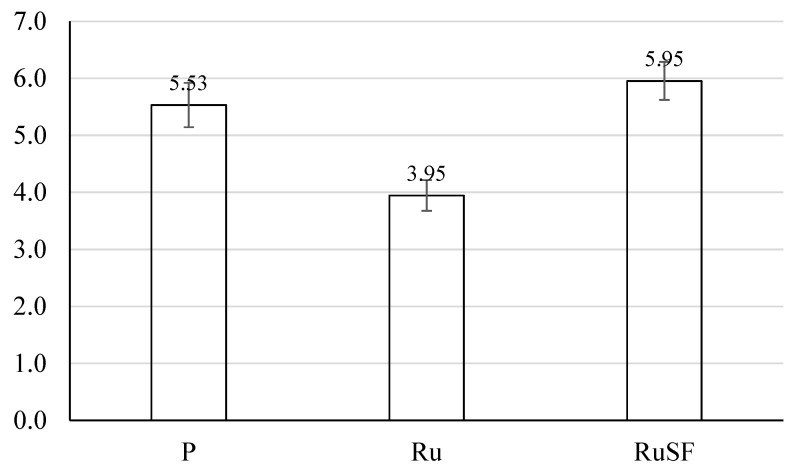
Splitting tensile strength of all mixes.

The average splitting tensile strength values are presented in Figure 6 for the different concrete mixes. The strength was 5.53 MPa for the plain concrete mix; however, the presence of rubber aggregate decreased the plain concrete tensile strength by approximately 28.6%. This was also aligned with the compressive strength reduction caused by the rubber addition, but to a lesser extent (i.e., 44.2% vs. 28.6%). Combining the RuC with steel fibers (mix RuSF) resulted in a splitting tensile strength 7.6% higher than for the plain mix. This indicates that blending RuC with steel fibers can at least recover the reduction in the tensile strength exhibited by rubber addition. However, an additional increase in the content of steel fibers, although it generally improves the tensile strength of the mix, reduces the mix workability. The reduced mix workability can introduce voids and hence results in relatively reduced mechanical properties. 

### 3.2. Flexure Behavior of the Beams

#### 3.2.1. Cracking Behavior and Mode of Failure

**Figure 7:**
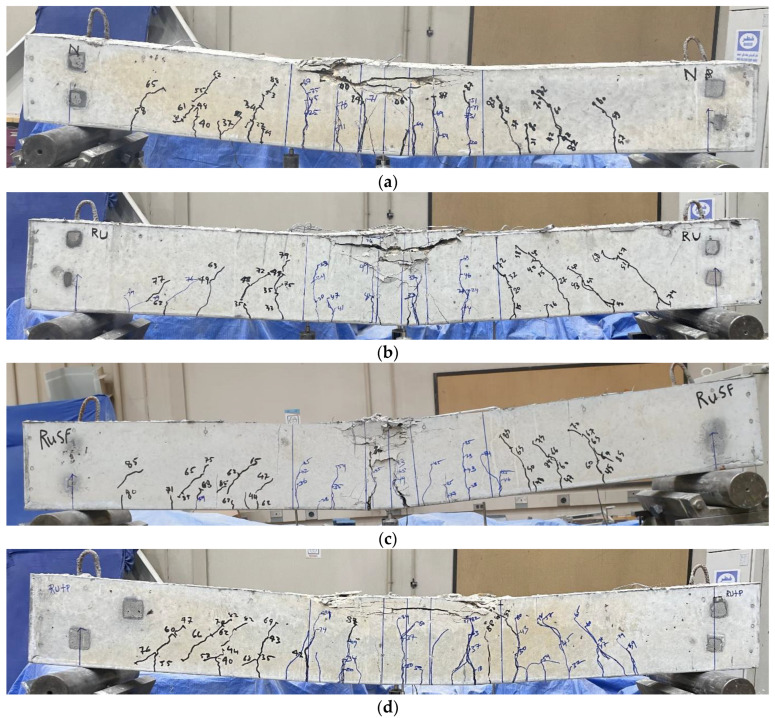

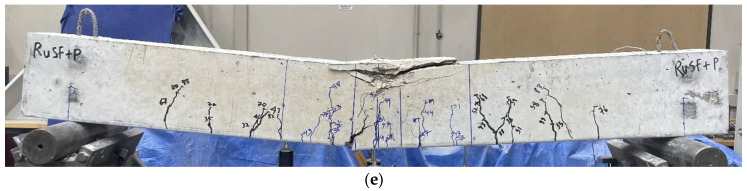
Cracking patterns and failure modes for (**a**) P; (**b**) Ru; (**c**) RuSF; (**d**) Ru + P; and (**e**) RuSF + P beams.

The cracking patterns and mode of failure of the five beams are provided in Figure 7. In Figure 7a, the plain concrete beam showed a typical flexural behavior, as intended in the design. The initial observable flexural crack was detected when the load reached 20 kN in the pure bending region at the beam mid-span. With increasing load, more flexural cracks were initiated around the mid-span region, then began to appear at the beam sides. At higher load levels, existing flexural cracks either extended vertically or diagonally to form flexural shear cracks. The final failure mode was characterized by pure flexural failure, as indicated by the concrete crushing at the top of the beam mid-span. The peak load was attained at 88.9 kN. For the rubberized beam Ru, the flexural behavior and cracking patterns were generally similar to those of the plain concrete beam (Figure 7b), although the initial observable crack was detected at a lower load of approximately 15 kN. Additionally, the extent of damage in terms of depth for the crushed concrete in the compressive area at the beam mid-span was relatively greater than the plain concrete beam, with a lower flexural strength of 79.5 kN. The cracking behavior observed in the rubberized beam with steel fibers (RuSF) exhibited a typical flexural failure similar to beams P and Ru, but the inclusion of the steel fibers delayed the appearance of the first visible flexural crack until a load of 28 kN was reached, with reduced crack width. The ultimate strength of this beam was 85.5 kN.

The overall behavior of the FG beam Ru + P (Figure 7d) was similar to that of the rubberized beam in the bottom zone, and to the plain beam in the top zone. Owing to the improved compressive strength in the compression area exhibited by the plain concrete, the depth of damage within the beam’s compressive zone resembled that of the plain concrete beam. Similarly, the weaker tensile strength of the RuC caused the initial observable crack to occur at a load of 18 kN, which is less than for the plain concrete beam. This observation is in line with the findings of Sharaky et al. [38] for the FG beam with bottom and top parts cast with rubberized and plain concrete, respectively, (B3M3T0) vs. the plain beam (CB), where the cracking load for the beam B3M3T0 was less than the plain beam. 

The behavior of the FG beam RuSF + P (Figure 7e) was also a combination of the lower part behavior of the SFRRuC and the upper part behavior of the plain concrete in terms of the density, the width and extension of cracks, and damage to the compressive zone. The first visible crack for this beam was detected at approximately 27 kN, which is close to the load at which the first crack appeared for the beam RuSF. Furthermore, the failure load for beams Ru + P and RuSF + P approximated or exceeded the plain beam failure load.

#### 3.2.2. Load–Deflection and Longitudinal Strain Response

**Figure 8:**
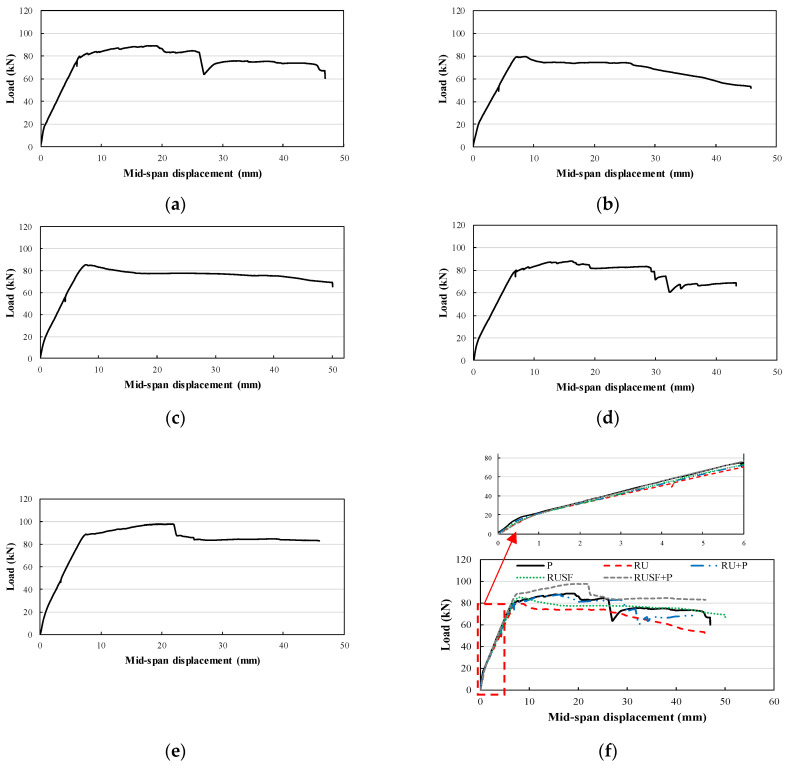
Force–deflection relationship for (**a**) P; (**b**) Ru; (**c**) RuSF; (**d**) Ru + P; (**e**) RuSF + P; and (**f**) all beams.

The load–deflection response of the different beams along with their comparisons are presented in Figure 8, in which the typical pre-peak responses for all beams are linear until the cracking load is reached, which is then followed by a linear relationship but with reduced stiffness up to the peak load. It can be seen from the various curves that the post-peak responses indicate that the beams experienced typical flexural failures. The only limited difference is noted in the initial stiffness of the various beams. The beam cast with only RuC (RU) had the lowest stiffness. Adding steel fibers to the RuC beam (RuSF) improved the initial stiffness, but did not reach the plain beam’s (P) stiffness. 

In Figure 8, the stiffness of the FG beam Ru + P is similar to that of beam RuSF. However, the initial stiffness of FG beam RuSF + P is equivalent to that of the plain beam. Clearly, the rubber addition reduced flexural stiffness, but both the incorporation of steel fibers and/or the adoption of FG beams (with the top layer of plain concrete) partially or fully recovered the reduction.

**Figure 9:**
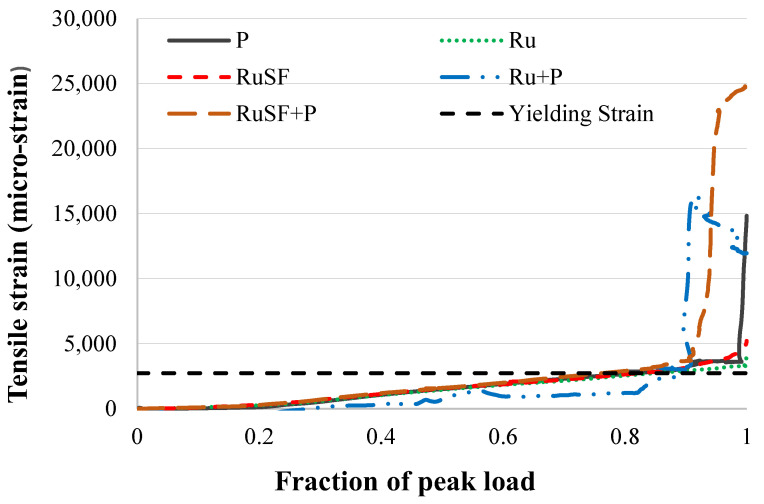
Comparisons of longitudinal steel tensile strain at different load levels for all beams.

The tensile strains developed in the longitudinal steel at different loading stages up to the ultimate load are presented in Figure 9. As depicted in the figure, all beams experienced yielding prior to reaching their ultimate flexural strength. The tensile steel of beams P, Ru, RuSF, Ru + P, and RuSF + P yielded at 0.82, 0.85, 0.82, 0.90, and 0.76 of their peak load, respectively. The strain observed at the ultimate flexural strength varied considerably. For example, the tensile steel in beams with a single layer of either plain, RuC, or SFRRuC developed tensile strains of 14,840, 4125, and 5217 micro-strain, respectively, but steel in the FG beams developed strains that were relatively smaller or larger than the plain concrete beam, recording 11,961 micro-strain for beam Ru + P and 25,229 micro-strain for beam RuSF + P. The reduced strains for beams with the full section cast with rubberized or steel reinforced RuC are explained by the lowered compressive strength of the RuC, resulting in lower resisting tensile forces.

#### 3.2.3. Effect of RuC on the Flexural Response of Concrete Beams

**Figure 10:**
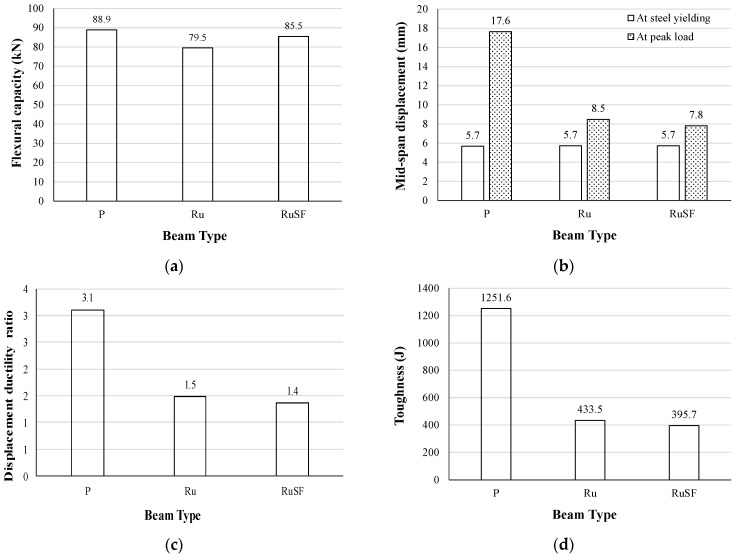
Effect of RuC beams with and without steel fibers on (**a**) flexural capacity; (**b**) mid-span displacement; (**c**) displacement ductility ratio; and (**d**) toughness.

The flexural responses (flexural capacity, mid-span displacement, displacement ductility ratio, and toughness) are compared in Figure 10 for the beams cast with only one material, i.e., plain concrete, RuC, and RuC with steel fibers. Figure 10a shows that utilizing RuC across the whole cross-section of the beam caused a 10.6% reduction in flexural strength relative to the beam with no rubber. However, combining the rubber particles with a blend of 40 kg/m^3^ of steel fibers led to a drop of only 3.8% in reference to the beam with no rubber. Such a decrease in flexural capacity is aligned with the drop in compressive strength exhibited by the rubber addition with or without steel fibers. However, the rate of reduction for the flexural strength is much lower than that for compressive strength.

The mid-span displacements at the yield point of the steel and at peak load are given in Figure 10b. It is evident that all beams reached the same displacement of 5.7 mm at the steel yield point. However, the mid-span displacement at peak load was significantly lower with the addition of rubber: in detail, the displacement decreased from 17.6 mm for the plain (P) beam to 8.5 mm and 7.8 mm for rubberized (Ru) and steel fiber reinforced rubberized (RuSF) concrete beams, respectively. This is consistent with the tensile strains observed for those beams, as the beam with plain concrete developed much higher plastic strains than the other beams. The displacement ductility, calculated as the ratio of mid-span displacement corresponding to the peak load to the displacement at steel yield point, is shown in Figure 10c. Similarly, the beam toughness obtained as the area underneath the load–deflection curve is illustrated in Figure 10d. As a consequence of the reduced displacement at peak load, both the displacement ductility and the toughness were reduced, with the incorporation of rubber with or without steel fibers following a similar trend. The decrease in the ductility ratio and toughness are consistent with the experimental observations of Ismail and Hassan [41]. It was observed that substituting fine aggregates with up to 20% crumb rubber improved the ductility and toughness, while any higher rate of rubber replacement reduced them due to the weakened concrete in the compression zone. It is crucial to highlight that in this study, both coarse and fine aggregates were each substituted with 20% rubber, which should be similar to the trend obtained by replacing either coarse or fine aggregate by more than 20%.

#### 3.2.4. Effectiveness of Utilizing Functionally Graded Beams

**Figure 11:**
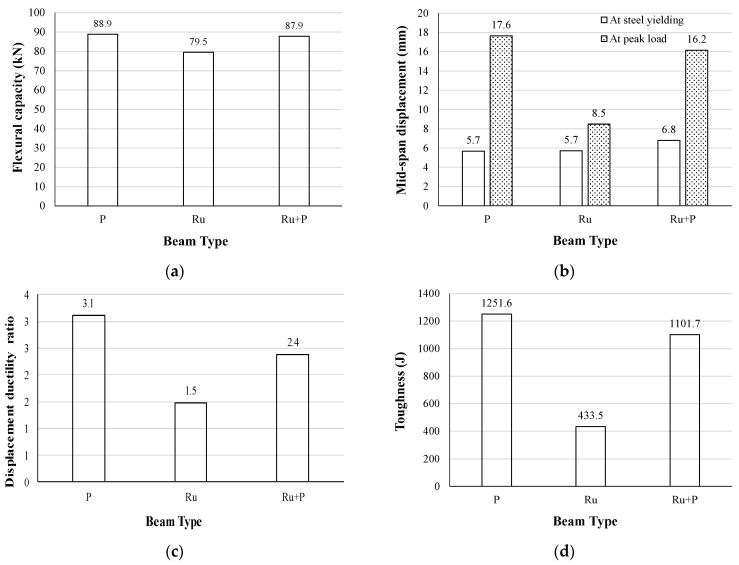
Effect of FG RuC beams on (**a**) flexural capacity; (**b**) mid-span displacement; (**c**) displacement ductility ratio; and (**d**) toughness.

**Figure 12:**
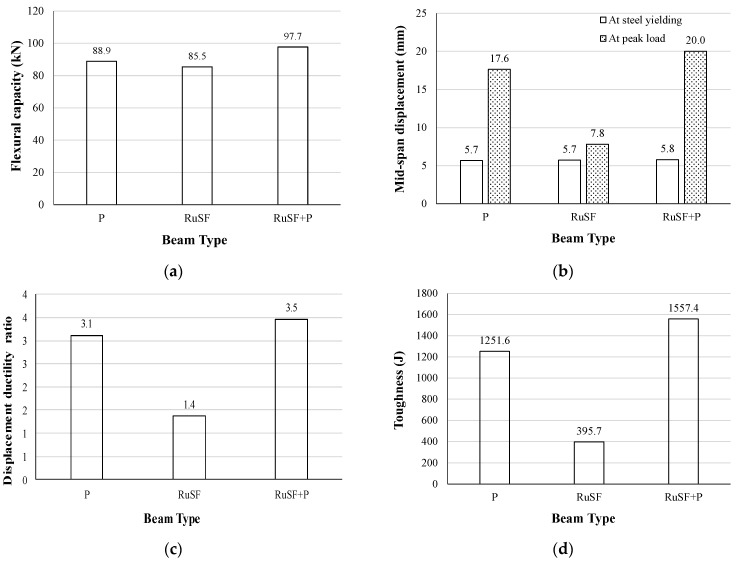
Effect of FG SFRRuC beams on (**a**) flexural capacity; (**b**) mid-span displacement; (**c**) displacement ductility ratio; and (**d**) toughness.

The influence on the flexural behavior of employing two materials across the beam depth in the cases of RuC beams and SFRRuC beams is demonstrated in Figure 11 and Figure 12, respectively. For the rubberized beams without steel fibers, Figure 11a shows that, although the flexural capacity was reduced by 10.6% in the beam with fully RuC, combining the plain concrete with the RuC within the same beam cross-section successfully recovered the flexural capacity to approximately 98.9% of the plain beam capacity. This improvement in flexural capacity is caused by the substitution of the relatively weak RuC in the compression area with the higher-strength plain concrete, thus increasing the compressive resistance; however, the beam tensile resistance is mostly controlled by the steel reinforcement.

The mid-span deflection at peak load for the FG beam was 90.6% higher than the displacement of the fully rubberized beam and very closely approached the displacement of the plain beam (16.2 mm vs. 17.6 mm), as shown in Figure 11b. This improvement could be justified by the improved concrete compressive strength, which allows a greater concrete strain capacity and, therefore, a higher balancing tensile force, leading to a development of higher tensile strains in the steel reinforcement. As a consequence of displacement capacity improvement, the displacement ductility ratio increased from 1.5 for the fully rubberized beam to 2.4 for the FG beam, thus recovering a significant portion of the lost ductility caused by the inclusion of a large quantity of rubber. Similarly, the beam toughness (i.e., the energy absorption capacity of the beam) was 1101.7 J for the FG beam, which was a significant enhancement on the fully rubberized beam toughness of 433.5 J (Figure 11d). The enhancement in toughness is related to the improvement in both the flexural capacity and displacement of the FG beam compared to the rubberized beam.

The observations made on the flexural strength and mid-span displacement at peak load for the plain beam (P) vs. the FG beam (Ru + P) were also consistent with research conducted previously by Sharaky et al. [38] for the plain beam (CB) vs. the FG beam (B3M3T0).

The FG beams combining a bottom layer of SFRRuC and a top layer of plain concrete exhibited excellent flexural behavior, as demonstrated by the flexural strength, mid-span displacement, ductility ratio, and toughness comparisons in Figure 12. Although the flexural capacity was not significantly reduced in the beam made entirely of SFRRuC (only 3.8% reduction) relative to the plain beam, the flexural capacity of the FG beam exceeded that of the plain beam by 9.9% (Figure 12a). Compared with the beam consisting of only SFRRuC, the FG beam RuSF + P not only overcame the degradation in flexural response, but the mid-span displacement at peak strength, displacement ductility ratio, and toughness outperformed the response of the plain beam by 13.6%, 12.9% and 24.4%, respectively.

From the above discussion, it is clear that the FG beam concept for RuC or SFRRuC is a very effective option, since it maximizes the utilization of waste rubber particles in relatively large quantities for concrete beams. At the same time, RuC achieves a range of benefits without compromising the flexural response. By utilizing a bottom layer of RuC in FG beams, the response is similar, or close to that of plain beams. However, adding steel fibers to the RuC in the FG beams was found to produce a better flexural response than for plain beams. This study confirmed the possibility of a similar or better flexural response than plain beams being achieved by optimizing the cross-section materials, and needing no additional cement or supplementary cementitious materials.

## 4. Moment–Curvature Relationship

The moment curvature derived experimentally for the various beams generally yielded consistent observations with the force–displacement curves discussed above. The prediction of the moment–curvature relationship was developed from the sectional analysis in the following subsections, and was compared with the experimentally derived relationship.

### 4.1. Moment–Curvature Prediction

A theoretical nonlinear moment–curvature relationship capturing the response of the tested beams was developed utilizing a fiber element analysis approach through EXCEL spreadsheets. The proper establishment of such a relationship requires defining representative constitutive models for steel rebars and concrete. The model cited by Priestley et al. [42] was adopted to model the tensile and compressive stress–strain behavior of longitudinal steel. This model defines three regions: the elastic, strain hardening, and plastic regions.

The concrete compressive stress–strain behavior was adopted following the unconfined concrete model proposed by Mander et al. [43], as described in Equations (1)–(3):

(1) $$f_{c}=\frac{{f'}_{c}\;\left[\frac{\varepsilon_{c}}{\varepsilon_{c0}}\right]\;r}{r-1+{\left[\frac{\varepsilon_{c}}{\varepsilon_{c0}}\right]}^{r}}\;\;\;\;\;\;\;\;\;\varepsilon_{c}\leq {2\varepsilon }_{c0\;},$$

(2) $$f_{c}=\frac{{2f'}_{c}\;r}{r-1+2^{r}}-\left(\frac{\varepsilon_{sp-}\varepsilon_{c}}{\varepsilon_{sp-}2\varepsilon_{c0}}\right)\;\;\;\;\;\;\;\;\;\;\varepsilon_{sp}\geq \varepsilon_{c\;}>2\varepsilon_{c0},$$

(3) $$r=\frac{E_{c}}{E_{c}\;–\;\frac{{f'}_{c}}{\varepsilon_{c0}}},$$

where ${f'}_{c}$ is the unconfined concrete compressive stress at compressive strain $\varepsilon_{c}$; $\varepsilon_{c0}$ is the strain at peak strength; $\varepsilon_{sp}$ is the spalling strain, which was taken to be 0.005; and $E_{c}$ is the concrete modulus of elasticity. The modulus of elasticity was used for all types of concrete (plain concrete, RuC, and SFRRuC) based on the CSA A23.3 [44] model given in Equation (4). The selection of this model was supported by the good prediction for all types of concrete used in this study relative to other models, including the model recommended in ACI 318 [45]. Similarly, the strain at peak strength was selected based on the model proposed by Tomaszewicz [46], as given by Equation (5). In fact, this model was selected because of its excellent prediction of strain at peak strength as compared to other available models. Importantly, the model predictions of both the elastic modulus and strain at peak strength were based solely on the compressive strength of concrete.

(4) $$f_{c}=4500\sqrt{{f'}_{c}\;}$$

(5) $$\varepsilon_{c0}=700\;{{f'}_{c}}^{0.31}\times 10^{-6}.$$

**Figure 13:**
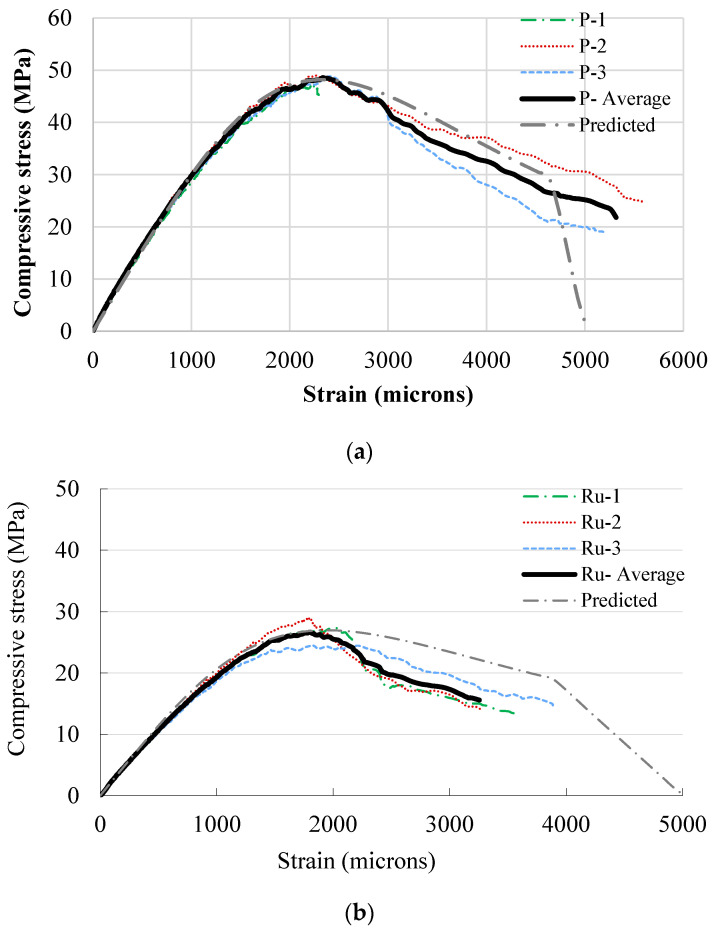

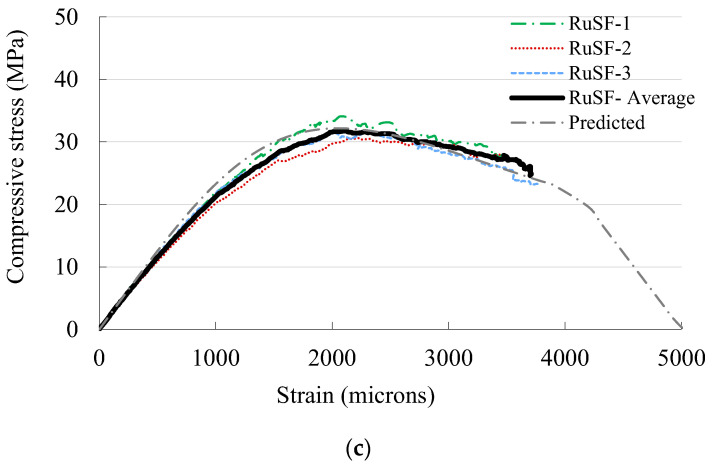
Compressive stress–strain validation for (**a**) P mix; (**b**) Ru mix; and (**c**) RuSF mix.

The predicted stress–strain relationship was validated against the observed experimental stress–strain relationships for the three types of concrete (Figure 13). The figure illustrates that the anticipated stress–strain relationship closely aligns with the experimental stress–strain relationship, despite some differences in the post-peak response of the RuC mix. Although many concrete compressive stress–strain models have been proposed in the literature for rubberized concrete [47] and steel fiber reinforced concrete [48]. The adopted model was selected due to its inherent simplicity, as its components are primarily linked to the compressive strength, which indirectly accounts for the variation in mix variables, including the rubber content and/or sizes. 

The tensile stress–strain relationship was modeled as suggested by Vecchio and Collins [49], described by Equations (6) and (7):

(6) $$f_{t}=E_{c}\;\varepsilon_{t}{\;\;\;\;\;\;\;\;\;\;\varepsilon }_{t\;}\leq \varepsilon_{cr}$$

(7) $$f_{t}=\frac{f_{cr}}{1+\sqrt{200\varepsilon_{t}\;}}\;\;\;\;\;\;\;\;\;\;\varepsilon_{t\;}>\varepsilon_{cr}$$

where $f_{t}$ is the concrete tensile stress at tensile strain $\varepsilon_{t}$; and $f_{cr}$ and $\varepsilon_{cr}$ are the cracking stress and strain of the concrete, respectively. For the plain concrete and RuC, the tensile cracking stress of the concrete was taken as the concrete modulus of rupture, as suggested by ACI 318 [50] and given by Equation (8). However, the relationship proposed by Aref et al. [51] was utilized to estimate the cracking stress for the SFRRuC, as given by Equation (9).

(8) $$f_{r}=0.62\sqrt{{f'}_{c}\;}$$

(9) $$f_{rFRC}=f_{r0}+2.246\;V_{f}\frac{l}{d\;}$$

where $f_{rFRC}$ is the modulus of rupture for steel fiber reinforced concrete; $f_{r0}$ is the tensile strength of concrete having no fibers; $V_{f}$ is the volume percentage of fibers; and $\frac{l}{d}$ is the aspect ratio of the steel fibers. The term $f_{r0}$ was calculated from Equation (8) and the compressive strength of RuC. Since the steel fibers were a blend of manufactured and recycled fibers, the aspect ratio for the manufactured steel fibers was adopted, since the aspect ratio of the recycled fibers varied greatly and could not be defined accurately.

### 4.2. Comparison of Predicted vs. Measured Moment Curvature 

**Figure 14:**
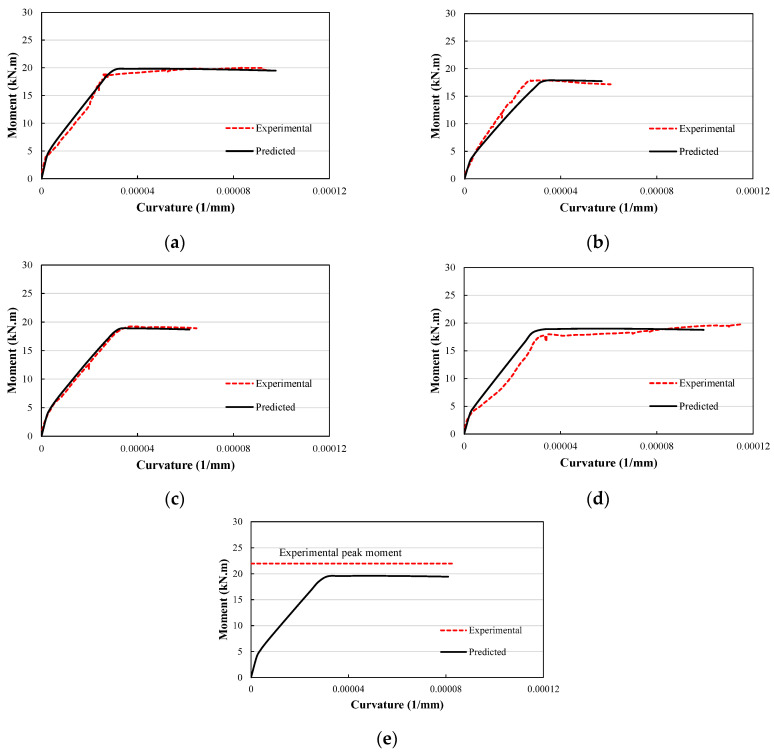
Predicted vs. experimental moment curvature for (**a**) P; (**b**) Ru; (**c**) RuSF; (**d**) Ru + P; and (**e**) RuSF + P.

The predicted vs. measured moment–curvature relationships for the investigated beams are given in Figure 14. It is seen in Figure 14e that only the measured peak moment was used to validate the predicted moment–curvature analysis for the beam RuSF + P, as the measured curvature values were unreliable due to an issue with the lateral LVDT measurements. Both the predicted and measured moment–curvatures are shown up to a point corresponding to a concrete compressive strain of 0.003 for all beams except the beam Ru + P, for which the moment–curvature relationship was predicted up to a point corresponding to a concrete compressive strain of 0.0035 to ensure the appearance of the peak moment.

It is seen in Figure 14 that the predicted moment–curvature relationships fit the experimental observations well in all the elastic and inelastic zones. In particular, the ratios of predicated ultimate moment to the experimental values for beams P, Ru, RuSF, Ru + P, and RuSF + P were 99.2%, 99.9%, 98.2%, 96.1%, and 89.1%, respectively.

## 5. Conclusions

This investigation included the testing of five beams under a four-point load configuration until failure to assess their flexural behavior. Control and functionally graded beams were cast with three types of concrete: plain concrete, rubberized concrete (RuC), and steel fiber reinforced rubberized concrete (SFRRuC). The rubberized beams were made by substituting 20% of the coarse and 20% of the fine natural aggregates with recycled rubber recovered from waste tires. The subsequent conclusions were derived:

- The first visible flexural crack (i.e., at the cracking moment) for the beam with only RuC appeared at a smaller load than the plain beam containing no rubber. However, the addition of steel fibers to the RuC postponed the occurrence of the initial flexural crack to an extent, exceeding the corresponding load for the plain beam. A similar conclusion was observed for the functionally graded beams, controlled by the bottom concrete layer of either RuC or SFRRuC.
- Despite the marginal reduction in initial flexural stiffness for the RuC, either the inclusion of steel fibers or the adoption of functionally graded beams (with an upper layer of plain concrete) partially or fully recovered the decrease in stiffness.
- Utilizing RuC across the whole cross-section of the beam resulted in a 10.6% lower flexural strength than the beam with no rubber. However, combining recycled rubber with 40 kg/m^3^ steel fibers enhanced the flexural strength, which was then reduced by only 3.8%. Furthermore, the mid-span deflection at peak load was found to decrease by approximately 51.7% and 55.7% for beams of only RuC and SFRRuC, respectively. As a consequence of the reduced displacement at peak load, both the displacement ductility and toughness were reduced with the incorporation of rubber with or without steel fibers.
- The flexural response of rubberized beams including or excluding steel fibers was significantly improved by adopting the functionally graded beams concept.
- The flexural behavior of the functionally graded beam with layers of RuC and plain concrete was superior to the fully rubberized beam, and closely approximated that of the plain beam in terms of flexural strength, displacement ductility ratio, and toughness. However, the flexural behavior of the functionally graded beam with a layer of SFRRuC and a layer of plain concrete exceeded the performance of the plain concrete beam.
- The moment–curvature relationships were predicted for the investigated beams and showed an excellent match with the experimentally measured relationships.

The outcome of this study revealed that large quantities of rubber particles can be incorporated into concrete mixtures used for casting beams through the concept of FGMs. In other words, utilizing FGMs would allow the exploitation of all the merits of rubberized concrete without compromising the flexural capacity of beams. In light of this research’s outcomes, future research on FGMs can be conducted to optimize the depth and quantity of rubber and or steel fibers with respect to the various flexural behavior components. 

## Figures and Tables

**Table 1:** Mix proportions for the various concrete mixes (kg/m^3^).

Ingredient/Mix ID.		P	Ru	RuSF
Cement		350	350	350
**Natural aggregate**	Red sand (0–1 mm)	560	448	448
	Crushed (1–5 mm)	240	192	192
	Coarse (5–10 mm)	330	264	264
	Coarse (10–20 mm)	715	572	572
**Rubber aggregate**	Fine (0–1 mm)	0	29.2	29.2
	Fine (1–5 mm)	0	12.5	12.5
	Coarse (5–10 mm)	0	18	18
	Coarse (10–20 mm)	0	39	39
Free water		140	140	140
Superplasticizer (PCE 575) (L)		2.8	3.5	5.25
Recycled tire steel fibers		0	0	20
Manufactured steel fibers		0	0	20

**Table 2:** Mechanical characteristics of steel reinforcement.

Property	Bar Diameter	
	8 mm	12 mm
Yield strength (MPa)	518.3	550.2
Ultimate strength (MPa)	528.8	651.2
Elastic modulus (GPa)	190	201.4

**Table 3:** Details of the investigated beams.

Designation	Beam Geometry (mm)			Stirrups (Dia. @ Spacing)	Flexural Steel		Concrete Mix	
	L	D	W		Top	Bottom	Layer 1 ^a^	Layer 2 ^b^
P	1500	180	120	8 @ 100 mm	Two 8 mm	Two 12 mm	P	
Ru	1500	180	120	8 @ 100 mm	Two 8 mm	Two 12 mm	Ru	
RuSF	1500	180	120	8 @ 100 mm	Two 8 mm	Two 12 mm	RuSF	
Ru + P	1500	180	120	8 @ 100 mm	Two 8 mm	Two 12 mm	Ru	P
RuSF + P	1500	180	120	8 @ 100 mm	Two 8 mm	Two 12 mm	RuSF	P

^a^ Layer 1 thickness is 60% from the beam depth measured from the bottom. ^b^ Layer 2 thickness is 40% from the beam depth measured from the top.

**Table 4:** Stress–strain components for the adopted mixes.

Mix	Compressive Strength (MPa)				Modulus of Elasticity (GPa)				Strain at Peak Strength (Micro-Strain)			
	1	2	3	Average	1	2	3	Average	1	2	3	Average
**P**	47.5	49.0	48.8	48.4	30.0	32.3	31.0	31.1	2245	2265	2390	2300.0
**Ru**	27.4	29.0	24.5	27.0	21.7	21.2	20.4	21.1	2040	1790	1820	1883.3
**RuSF**	34.1	30.6	31.8	32.2	22.9	21.4	23.6	22.6	2085	2240	2015	2113.3

## Data Availability

Data are contained within the article.
